# Monotonicity rule for the quotient of two functions and its application

**DOI:** 10.1186/s13660-017-1383-2

**Published:** 2017-05-09

**Authors:** Zhen-Hang Yang, Wei-Mao Qian, Yu-Ming Chu, Wen Zhang

**Affiliations:** 1 Department of Mathematics, Huzhou University, Huzhou, 313000 China; 2 School of Distance Education, Huzhou Broadcast and TV University, Huzhou, 313000 China; 3 Friedman Brain Institute, Icahn School of Medicine at Mount Sinai, New York, 10029 USA

**Keywords:** 26A48, 33E05, monotonicity rule, complete elliptic integral, absolute error, relative error

## Abstract

In the article, we provide a monotonicity rule for the function $[P(x)+A(x)]/[P(x)+B(x)]$, where $P(x)$ is a positive differentiable and decreasing function defined on $(-R, R)$ ($R>0$), and $A(x)=\sum ^{\infty}_{n=n_{0}}a_{n}x^{n}$ and $B(x)=\sum^{\infty }_{n=n_{0}}b_{n}x^{n}$ are two real power series converging on $(-R, R)$ such that the sequence $\{a_{n}/b_{n}\}_{n=n_{0}}^{\infty}$ is increasing (decreasing) with $a_{n_{0}}/b_{n_{0}}\geq(\leq)\ 1$ and $b_{n}>0$ for all $n\geq n_{0}$. As applications, we present new bounds for the complete elliptic integral $\mathcal{E}(r)=\int_{0}^{\pi /2}\sqrt{1-r^{2}\sin^{2}t}\,dt$ ($0< r<1$) of the second kind.

## Introduction

The most commonly used monotonicity rule in elementary calculus is that *f* is increasing (decreasing) on $[a, b]$ if $f: [a, b]\rightarrow \mathbb{R}$ is continuous on $[a, b]$ and has a positive (negative) derivative on $(a, b)$, and it can be proved easily by the Lagrange mean value theorem. The functions whose monotonicity we prove in this way are usually polynomials, rational functions, or other elementary functions. But we often find that the derivative of a quotient of two functions is quite messy and the process is tedious. Therefore, the improvements, generalizations and refinements of the method for proving monotonicity of quotients have attracted the attention of many researchers.

In 1955, Biernacki and Krzyż [1] (see also [2], Lemma 2.1, [3]) found an important criterion for the monotonicity of the quotient of power series as follows.

### Theorem 1.1

[1]

*Let* $A(t)=\sum_{k=0}^{\infty }a_{k}t^{k}$ *and* $B(t)=\sum_{k=0}^{\infty}b_{k}t^{k}$ *be two real power series converging on* $(-r,r)$ ($r>0$) *with* $b_{k}>0$ *for all* *k*. *If the non*-*constant sequence* $\{a_{k}/b_{k}\}$ *is increasing* (*decreasing*) *for all* *k*, *then the function* $t\mapsto A(t)/B(t)$ *is strictly increasing* (*decreasing*) *on* $(0,r)$.

In [4], Cheeger et al. presented the monotonicity rule for the quotient of two functions.

### Theorem 1.2

[4]

*If* *f* *and* *g* *are positive integrable functions on* $\mathbb{R}$ *such that* $f/g$ *is decreasing*, *then the function* $x\mapsto\int_{0}^{x}f(t)\,dt/\int_{0}^{x}g(t)\, dt$ *is decreasing*.

Unaware of Theorem 1.2, Anderson et al. [5], Lemma 2.2 (see also [6], Theorem 1.25) established l’Hôpital’s monotone rule that can be applied to a wide class of quotients of functions.

### Theorem 1.3

[5]

*Let* $-\infty< a< b<\infty$, $f,g:[a,b]\rightarrow{\mathbb{R}}$ *be continuous on* $[a,b]$ *and differentiable on* $(a,b)$, *and* $g'(x)\neq0$ *on* $(a,b)$. *If* $f^{\prime}(x)/g^{\prime}(x)$ *is increasing* (*decreasing*) *on* $(a,b)$, *then so are the functions*

$$ \frac{f(x)-f(a)}{g(x)-g(a)}, \qquad\frac{f(x)-f(b)}{g(x)-g(b)}. $$

*If* $f^{\prime}(x)/g^{\prime}(x)$ *is strictly monotone*, *then the monotonicity in the conclusion is also strict*.

Pinelis [7] provided the following monotonicity theorem.

### Theorem 1.4

[7]

*Let* *f* *and* *g* *be differentiable and* $g^{\prime}$ *never vanish on an open interval* $(a, b)\subset\mathbb{R}$. *Then the following statements are true*:

1. *If* $gg^{\prime}>0$ *on* $(a,b)$, $\lim\sup_{x\downarrow a}g^{2}(x)(f(x)/g(x))^{\prime}/|g^{\prime}(x)|\geq0$ *and* $f^{\prime }/g^{\prime}$ *is increasing on* $(a,b)$, *then* $(f/g)^{\prime}>0$ *on* $(a,b)$.
2. *If* $gg^{\prime}>0$ *on* $(a,b)$, $\lim\inf_{x\downarrow a}g^{2}(x)(f(x)/g(x))^{\prime}/|g^{\prime}(x)|\leq0$ *and* $f^{\prime }/g^{\prime}$ *is decreasing on* $(a,b)$, *then* $(f/g)^{\prime}<0$ *on* $(a,b)$.
3. *If* $gg^{\prime}<0$ *on* $(a,b)$, $\lim\inf_{x\uparrow b}g^{2}(x)(f(x)/g(x))^{\prime}/|g^{\prime}(x)|\leq0$ *and* $f^{\prime }/g^{\prime}$ *is increasing on* $(a,b)$, *then* $(f/g)^{\prime}<0$ *on* $(a,b)$.
4. *If* $gg^{\prime}<0$ *on* $(a,b)$, $\lim\sup_{x\uparrow b}g^{2}(x)(f(x)/g(x))^{\prime}/|g^{\prime}(x)|\geq0$ *and* $f^{\prime }/g^{\prime}$ *is increasing on* $(a,b)$, *then* $(f/g)^{\prime}>0$ *on* $(a,b)$.

Recently, Yang et al. [8], Theorem 1.2, established a more general monotonicity rule for the ratio of two power series.

### Theorem 1.5

[8]

*Let* $A(t)=\sum_{k=0}^{\infty}a_{k}t^{k}$ *and* $B(t)=\sum_{k=0}^{\infty}b_{k}t^{k}$ *be two real power series converging on* $(-r,r) $ *and* $b_{k}>0$ *for all* *k*, *and* $H_{A, B}=A^{\prime}B/B^{\prime}-A$. *Suppose that for certain* $m\in\mathbb{N}$, *the non*-*constant sequence* $\{a_{k}/b_{k}\}$ *is increasing* (*decreasing*) *for* $0\leq k\leq m$ *and decreasing* (*increasing*) *for* $k\geq m$. *Then the function* $A/B$ *is strictly increasing* (*decreasing*) *on* $(0,r) $ *if and only if* $H_{A,B}(r^{-}) \geq(\leq)\ 0$. *Moreover*, *if* $H_{A,B}(r^{-})<(>) 0$, *then there exists* $t_{0}\in(0,r) $ *such that the function* $A/B$ *is strictly increasing* (*decreasing*) *on* $(0,t_{0}) $ *and strictly decreasing* (*increasing*) *on* $(t_{0},r)$.

The foregoing monotonicity rules have been used very effectively in the study of special functions [9–23], differential geometry [4, 24], probability [25] and approximation theory [26]. The main purpose of the article is to present the monotonicity rule for the function $[P(x)+\sum_{n=n_{0}}^{\infty}a_{n}x^{n}]/[P(x)+\sum _{n=n_{0}}^{\infty}b_{n}x^{n}]$ and to provide new bounds for the complete elliptic integral of the second kind. Some complicated computations are carried out using Mathematica computer algebra system.

## Monotonicity rule

### Theorem 2.1

*Let* $P(x)$ *be a positive differentiable and decreasing function defined on* $(0, r)$ ($r>0$), *let* $A(x)=\sum _{n=n_{0}}^{\infty}a_{n}x^{n}$ *and* $B(x)=\sum_{n=n_{0}}^{\infty}b_{n}x^{n}$ *be two real power series converging on* $(-r, r)$. *If* $a_{n_{0}}/b_{n_{0}}\geq(\leq)\ 1$, $b_{n}>0$ *for all* $n\geq n_{0}$ *and the non*-*constant sequence* $\{ a_{n}/b_{n}\}_{n=n_{0}}^{\infty}$ *is increasing* (*decreasing*), *then the function* $x\mapsto[P(x)+A(x)]/[P(x)+B(x)]$ *is strictly increasing* (*decreasing*) *on* $(0, r)$.

### Proof

Let $x\in(0, r)$, and

2.1 $$ \begin{aligned} &I_{1}=\sum _{n=n_{0}}^{\infty}\bigl[nP(x)-xP^{\prime}(x) \bigr](a_{n}-b_{n})x^{n-1}, \\ &I_{2}=\sum_{n=n_{0}}^{\infty}na_{n}x^{n-1} \sum_{n=n_{0}}^{\infty}b_{n}x^{n} -\sum_{n=n_{0}}^{\infty}a_{n}x^{n} \sum_{n=n_{0}}^{\infty}nb_{n}x^{n-1}. \end{aligned} $$

Then differentiating $[P(x)+A(x)]/[P(x)+B(x)]$ gives

2.2 $$\begin{aligned} & \bigl[ P(x)+B(x) \bigr] ^{2} \biggl[ \frac{P(x)+A(x)}{P(x)+B(x)} \biggr] ^{\prime} \\ &\quad= \Biggl( P^{\prime}(x)+\sum_{n=n_{0}}^{\infty}na_{n}x^{n-1} \Biggr) \Biggl( P(x)+\sum_{n=n_{0}}^{\infty}b_{n}x^{n} \Biggr) \\ &\quad\quad{}- \Biggl( P(x)+\sum_{n=n_{0}}^{\infty}a_{n}x^{n} \Biggr) \Biggl( P^{\prime}(x)+\sum_{n=n_{0}}^{\infty}nb_{n}x^{n-1} \Biggr) \\ &\quad=I_{1}+I_{2}. \end{aligned}$$

Note that $I_{2}$ can be rewritten as

2.3 $$\begin{aligned} I_{2}={}&\sum_{j=n_{0}}^{\infty}ja_{j}x^{j-1} \sum_{i=n_{0}}^{\infty}b_{i}x^{i}- \sum_{i=n_{0}}^{\infty}a_{i}x^{i} \sum_{j=n_{0}}^{\infty}jb_{j}x^{j-1} \\ ={}&\sum_{i=n_{0}}^{\infty}\sum _{j=n_{0}}^{\infty}jb_{i}b_{j} \biggl( \frac{a_{j}}{b_{j}}-\frac{a_{i}}{b_{i}} \biggr) x^{i+j-1} \\ ={}&\sum _{j=n_{0}}^{\infty}\sum_{i=n_{0}}^{\infty}ib_{j}b_{i} \biggl( \frac{a_{i}}{b_{i}}-\frac{a_{j}}{b_{j}} \biggr) x^{i+j-1} \\ ={}&\frac{1}{2}\sum_{i=n_{0}}^{\infty}\sum _{j=n_{0}}^{\infty}b_{i}b_{j}(i-j) \biggl( \frac{a_{i}}{b_{i}}-\frac{a_{j}}{b_{j}} \biggr) x^{i+j-1}. \end{aligned}$$

If $a_{n_{0}}/b_{n_{0}}\geq(\leq)\ 1$, $b_{n}>0$ for all $n\geq n_{0}$ and the non-constant sequence $\{a_{n}/b_{n}\}_{n=n_{0}}^{\infty }$ is increasing (decreasing), then we clearly see that

2.4 $$ a_{n}\geq(\leq)\ b_{n} $$

for all $n\geq n_{0}$ and

2.5 $$ \sum_{i=n_{0}}^{\infty}\sum _{j=n_{0}}^{\infty}b_{i}b_{j}(i-j) \biggl( \frac{a_{i}}{b_{i}}-\frac{a_{j}}{b_{j}} \biggr) x^{i+j-1}>(< )\ 0 $$

for all $x\in(0, r)$.

It follows from $P(x)$ is a positive differentiable and decreasing function on $(0, r)$ that

2.6 $$ nP(x)-xP^{\prime}(x)>0 $$

for all $x\in(0, r)$.

Therefore, $[(P(x)+A(x))/(P(x)+B(x))]^{\prime}>$ (<) 0 for all $x\in(0, r)$ follows easily from (2.1)-(2.6), and the proof of Theorem 2.1 is completed. □

## Bounds for the complete elliptic integral of the second kind

For $r\in(0,1)$, Legendre’s complete elliptic integral [27] of the second kind is given by

$$ \mathcal{E}(r)= \int_{0}^{\pi/2}\sqrt{1-r^{2}\sin ^{2}(t)}\,dt. $$

It is well known that $\mathcal{E}(0^{+})=\pi/2$, $\mathcal {E}(1^{-})=1$, and $\mathcal{E}(r)$ is the particular case of the Gaussian hypergeometric function

$$ F(a,b;c;x)=\sum_{n=0}^{\infty}\frac{(a)_{n}(b)_{n}}{(c)_{n}} \frac{x^{n}}{n!}\quad(-1< x< 1), $$

where $(a)_{n}=\Gamma(a+n)/\Gamma(a)$ and $\Gamma(x)=\int_{0}^{\infty}t^{x-1}e^{-t}\,dt$ ($x>0$) is the gamma function. Indeed, we have

3.1 $$ \mathcal{E}(r)=\frac{\pi}{2}F \biggl( -\frac{1}{2}, \frac{1}{2};1;r^{2} \biggr) =\frac{\pi}{2}\sum _{n=0}^{\infty}\frac{ ( -\frac{1}{2} ) _{n} ( \frac{1}{2} ) _{n}}{(n!)^{2}}r^{2n}. $$

Recently, the bounds for the complete elliptic integral $\mathcal {E}(r)$ of the second kind have been the subject of intensive research. In particular, many remarkable inequalities for $\mathcal{E}(r)$ can be found in the literature [28–41]. Vuorinen [42] conjectured that the inequality

3.2 $$ \mathcal{E}(r)\geq\frac{\pi}{2} \biggl( \frac {1+{r^{\prime}}^{3/2}}{2} \biggr) ^{2/3} $$

holds for all $r\in(0, 1)$, where, and in what follows, $r^{\prime }=(1-r^{2})^{1/2}$. Inequality (3.2) was proved by Barnard et al. in [43].

Very recently, the accurate bounds for $\mathcal{E}(r)$ in terms of the Stolarsky mean $S_{p, q}(1, r^{\prime})$ were given in [44, 45]:

3.3 $$\begin{aligned}& \frac{\pi}{2}S_{11/4, 7/4}\bigl(1, r^{\prime}\bigr)< \mathcal{E}(r)< \frac{11}{7}S_{11/4, 7/4}\bigl(1, r^{\prime}\bigr), \end{aligned}$$

3.4 $$\begin{aligned}& \frac{25}{16}S_{5/2, 2}\bigl(1, r^{\prime}\bigr)< \mathcal{E}(r)< \frac{\pi}{2}S_{5/2, 2}\bigl(1, r^{\prime}\bigr), \end{aligned}$$

where $S_{p, q}(a,b)=[q(a^{p}-b^{p})/(p(a^{q}-b^{q}))]^{1/(p-q)}$.

In this section, we shall use Theorem 2.1 to present new bounds for the complete elliptic integral $\mathcal{E}(r)$ of the second kind. In order to prove our main result, we need three lemmas, which we present in this section.

### Lemma 3.1

see [46], Lemma 7

*Let* $n\in\mathbb{N}$ *and* $m\in\mathbb{N}\cup\{0\}$ *with* $n>m$, $a_{i}\geq0$ *for all* $0\leq i\leq n$, $a_{n}a_{m}>0$ *and*

$$ P_{n}(t)=-\sum_{i=0}^{m}a_{i}t^{i}+ \sum_{i=m+1}^{n}a_{i}t^{i}. $$

*Then there exists* $t_{0}\in(0, \infty)$ *such that* $P_{n}(t_{0})=0$, $P_{n}(t)<0$ *for* $t\in(0, t_{0})$ *and* $P_{n}(t)>0$ *for* $t\in(t_{0}, \infty)$.

### Lemma 3.2

see [47, 48]

*The double inequality*

$$ \frac{1}{(x+a)^{1-a}}< \frac{\Gamma(x+a)}{\Gamma(x+1)}< \frac{1}{x^{1-a}} $$

*holds for all* $x>0$ *and* $a\in(0, 1)$.

### Lemma 3.3

*Let* $n\in\mathbb{N}$, $p_{1}(n)$, $p_{2}(n)$, $p_{3}(n)$ *and* $w_{n}$ *be defined by*

3.5 $$\begin{aligned}& p_{1}(n)=125n^{4}+1\text{,}482n^{3}+1\text{,}601n^{2}-7\text{,}532n+1\text{,}944, \end{aligned}$$

3.6 $$\begin{aligned}& p_{2}(n)=4\text{,}375n^{6}+213\text{,}247n^{5}+1\text{,}696\text{,}697n^{4}-4\text{,}433\text{,}167n^{3} \\& \qquad \quad {}-1\text{,}571\text{,}832n^{2}+4\text{,}662\text{,}360n-1\text{,}555\text{,}200, \end{aligned}$$

3.7 $$\begin{aligned}& p_{3}(n)=625n^{6}+22\text{,}583n^{5}-304\text{,}413n^{4}+234\text{,}181n^{3} \\& \qquad \quad {}+1\text{,}906\text{,}328n^{2}-2\text{,}918\text{,}064n+675\text{,}360, \end{aligned}$$

3.8 $$\begin{aligned}& w_{n}=-648np_{1}(n) \biggl( \frac{1}{4} \biggr) _{n-2}+p_{2}(n) \biggl( \frac{1}{2} \biggr) _{n-3}+\frac{5np_{3}(n)}{2n-5} \biggl( \frac{3}{4} \biggr) _{n-3}, \end{aligned}$$

*respectively*. *Then* $w_{n}\geq0$ *for all* $n\geq3$.

### Proof

Let $p_{0}(n)$, $p_{4}(n)$, $p_{5}(n)$, $\alpha_{n}$ and $\beta_{n}$ be defined by

3.9 $$\begin{aligned}& p_{0}(n)=625n^{4}-2\text{,}018n^{3}+3\text{,}985n^{2}-4\text{,}032n+1\text{,}080, \end{aligned}$$

3.10 $$\begin{aligned}& p_{4}(n)=875n^{7}-81\text{,}128n^{6}-2\text{,}341\text{,}894n^{5}-10\text{,}120\text{,}928n^{4}+19\text{,}839\text{,}719n^{3} \\& \qquad \quad {}-22\text{,}615\text{,}904n^{2}+73\text{,}667\text{,}340n-71\text{,}971\text{,}200, \end{aligned}$$

3.11 $$\begin{aligned}& p_{5}(n)=250n^{6}-1\text{,}834n^{5}+391\text{,}275n^{4}+975\text{,}000n^{3}-7\text{,}770\text{,}415n^{2} \\& \quad \qquad {}+7\text{,}980\text{,}844n+298\text{,}140, \end{aligned}$$

3.12 $$\begin{aligned}& \alpha_{n}=\frac{p_{4}(n)}{n(n+1)} \biggl( \frac{1}{2} \biggr) _{n-3}, \end{aligned}$$

3.13 $$\begin{aligned}& \beta_{n}=\frac{10(n-1)p_{5}(n)}{(2n-3)(2n-5)} \biggl( \frac{3}{4} \biggr)_{n-3}, \end{aligned}$$

respectively.

Then from (3.5)-(3.13) and elaborated computations we get

3.14 $$\begin{aligned}& w_{3}=w_{4}=w_{5}=w_{6}=0, \end{aligned}$$

3.15 $$\begin{aligned}& w_{7}=\frac{2\text{,}848\text{,}355}{16}>0, \end{aligned}$$

3.16 $$\begin{aligned}& \frac{w_{n+1}}{(n+1)p_{1}(n+1)}-\frac{(n-7/4)w_{n}}{np_{1}(n)} \\& \quad = \frac{p_{2}(n+1)}{(n+1)p_{1}(n+1)} \biggl( \frac{1}{2} \biggr) _{n-2}- \frac{(n-7/4)p_{2}(n)}{np_{1}(n)} \biggl( \frac{1}{2} \biggr) _{n-3} \\& \quad \quad {}+\frac{5p_{3}(n+1)}{(2n-3)p_{1}(n+1)} \biggl( \frac{3}{4} \biggr) _{n-2}- \frac{5(n-7/4)p_{3}(n)}{(2n-5)p_{1}(n)} \biggl( \frac{3}{4} \biggr) _{n-3} \\& \quad = \biggl[ \frac{(n-5/2)p_{2}(n+1)}{(n+1)p_{1}(n+1)}-\frac {(n-7/4)p_{2}(n)}{np_{1}(n)} \biggr] \biggl( \frac{1}{2} \biggr) _{n-3} \\& \quad \quad {}+ \biggl[ \frac{5(n-9/4)p_{3}(n+1)}{(2n-3)p_{1}(n+1)}-\frac {5(n-7/4)p_{3}(n)}{(2n-5)p_{1}(n)} \biggr] \biggl( \frac{3}{4} \biggr) _{n-3} \\& \quad = \frac{p_{0}(n+1)p_{4}(n)}{4n(n+1)p_{1}(n)p_{1}(n+1)} \biggl( \frac {1}{2} \biggr) _{n-3} \\& \quad \quad {}+\frac{5(n-1)p_{0}(n+1)p_{5}(n)}{2(2n-3)(2n-5)p_{1}(n)p_{1}(n+1)} \biggl( \frac{3}{4} \biggr) _{n-3} \\& \quad = \frac{p_{0}(n+1)}{4p_{1}(n)p_{1}(n+1)} ( \alpha_{n}+\beta_{n} ) , \end{aligned}$$

3.17 $$\begin{aligned}& \frac{\alpha_{n}}{\beta_{n}}=\frac {(2n-3)(2n-5)p_{4}(n)}{10n(n-1)(n+1)p_{5}(n)} \frac{ ( \frac{1}{2} ) _{n-3}}{ ( \frac{3}{4} ) _{n-3}}, \end{aligned}$$

3.18 $$\begin{aligned}& \frac{\alpha_{7}}{\beta_{7}}=-\frac{7\text{,}313\text{,}056}{7\text{,}313\text{,}875}, \end{aligned}$$

3.19 $$\begin{aligned}& \frac{10 ( \frac{3}{4} ) _{n-3}}{ ( \frac{1}{2} ) _{n-1}} \biggl( \frac{\alpha_{n+1}}{\beta_{n+1}}-\frac{\alpha_{n}}{\beta_{n}} \biggr) \\& \quad = \frac{(2n-1)(2n-3)(n-5/2)p_{4}(n+1)}{n(n+1)(n+2)(n-9/4)p_{5}(n+1)}-\frac{(2n-3)(2n-5)p_{4}(n)}{n(n-1)(n+1)p_{5}(n)} \\& \quad = -\frac{(2n-3)(2n-5)(n-3)(n-4) (125n^{4}+1\text{,}982n^{3}+6\text{,}797n^{2}+616n-2\text{,}380 )}{n(4n-9)(n-1)(n+1)(n+2)p_{5}(n)p_{5}(n+1)} \\& \quad \quad {} \times\bigl(1\text{,}750n^{8}-802\text{,}264n^{7}-54\text{,}353\text{,}513n^{6}+811\text{,}227\text{,}431n^{5}-4\text{,}748\text{,}597\text{,}075n^{4} \\& \quad \quad {}+21\text{,}568\text{,}863\text{,}989n^{3}-80\text{,}185\text{,}046\text{,}202n^{2} \\& \quad \quad {}+144\text{,}028\text{,}552\text{,}644n-85\text{,}225\text{,}499\text{,}160 \bigr) \\& \quad = -\frac{(2n-3)(2n-5)(n-3)(n-4) ( 125n^{4}+1\text{,}982n^{3}+6\text{,}797n^{2}+616n-2\text{,}380 ) }{n(4n-9)(n-1)(n+1)(n+2)p_{5}(n)p_{5}(n+1)} \\& \quad \quad {} \times\bigl[-420\text{,}331\text{,}641\text{,}120-714\text{,}515\text{,}222\text{,}844(n-7)-475\text{,}749\text{,}995\text{,}856(n-7)^{2} \\& \quad \quad {}-152\text{,}526\text{,}681\text{,}341(n-7)^{3}-25\text{,}642\text{,}525\text{,}865(n-7)^{4} \\& \quad \quad {}-2\text{,}263\text{,}535\text{,}771(n-7)^{5} \\& \quad \quad {}-91\text{,}263\text{,}449(n-7)^{6}-704\text{,}264(n-7)^{7}+1\text{,}750(n-7)^{8} \bigr]. \end{aligned}$$

From Lemma 3.1 and (3.19) together with the facts that $p_{5}(n)>0$ and $p_{5}(n+1)>0$ for $n\geq7$, we clearly see that there exists $n_{0}>7$ such that the sequence $\{\alpha_{n}/\beta _{n}\}_{n=7}^{\infty}$ is increasing for $7\leq n\leq n_{0}$ and decreasing for $n\geq n_{0}$, which implies that

3.20 $$ \frac{\alpha_{n}}{\beta_{n}}\geq \min \biggl\{ \frac{\alpha _{7}}{\beta_{7}}, \lim _{n\rightarrow\infty}\frac{\alpha_{n}}{\beta_{n}} \biggr\} . $$

It follows from Lemma 3.2 that

3.21 $$ \frac{\Gamma ( \frac{3}{4} ) ( n-\frac{5}{2} ) ^{-1/4}}{\Gamma ( \frac {1}{2} ) } < \frac{ ( \frac{1}{2} ) _{n-3}}{ ( \frac{3}{4} ) _{n-3}}=\frac{\Gamma ( \frac{3}{4} ) }{\Gamma ( \frac{1}{2} ) }\frac {\Gamma ( n-\frac{13}{4}+\frac{3}{4} ) }{\Gamma ( n-\frac{13}{4}+1 ) } < \frac{\Gamma ( \frac{3}{4} ) ( n-\frac{13}{4} ) ^{-1/4}}{\Gamma ( \frac{1}{2} ) } $$

for all $n\geq7$.

From (3.10), (3.11), (3.17), (3.18), (3.20) and (3.21) we get

3.22 $$ \frac{\alpha_{n}}{\beta_{n}}\geq\frac{\alpha_{7}}{\beta_{7}}>-1 $$

for all $n\geq7$.

Therefore, Lemma 3.3 follows easily from (3.14)-(3.16) and (3.22) together with the facts that $p_{1}(n)>0$, $p_{0}(n+1)>0$ and $p_{1}(n+1)>0$ for $n\geq7$. □

### Theorem 3.4

*The double inequality*

3.23 $$ \frac{40(\pi-2)}{29}J\bigl(r^{\prime}\bigr)-\frac{51\pi-160}{58}< \mathcal{E}(r)< \frac{\pi}{2}J\bigl(r^{\prime}\bigr) $$

*holds for all* $r\in(0, 1)$, *where*

3.24 $$ J\bigl(r^{\prime}\bigr)=\frac{51{r^{\prime}}^{2}+20r^{\prime}\sqrt {r^{\prime}}+50r^{\prime}+20\sqrt{r^{\prime}}+51}{16(5r^{\prime }+2\sqrt{r^{\prime}}+5)}. $$

### Proof

Let $r\in(0, 1)$, $x=r^{2}$, $P(x)$, $f_{1}(r)$, $f_{2}(r)$ and $F(r)$ be defined by

3.25 $$\begin{aligned}& P(x)=1\text{,}536-1\text{,}248x, \end{aligned}$$

3.26 $$\begin{aligned}& f_{1}(r)= 2\text{,}592\bigl(2-r^{2}\bigr) \bigl(1-r^{2} \bigr)^{3/4}+15\bigl(425r^{4}-624r^{2}+192\bigr) \bigl(1-r^{2}\bigr)^{1/2} \\& \quad \quad {}-50\bigl(r^{4}-96r^{2}+96\bigr) \bigl(1-r^{2} \bigr)^{1/4}-105r^{4}+3\text{,}600r^{2}+2\text{,}880, \end{aligned}$$

3.27 $$\begin{aligned}& f_{2}(r)=625r^{4}-384r^{2}+384, \end{aligned}$$

3.28 $$\begin{aligned}& F(r)=\frac{1-J(r^{\prime})}{1-2\mathcal{E}(r)/\pi}, \end{aligned}$$

respectively.

Then from (3.1) and (3.24)-(3.28) we have

$$\begin{aligned}& f_{1}(r)= 2\text{,}880+3\text{,}600r^{2}-105r^{4}+2\text{,}592 \Biggl( 2 \sum_{n=0}^{\infty}\frac{ ( -\frac{3}{4} ) _{n}}{n!}r^{2n}- \sum_{n=1}^{\infty}\frac{ ( -\frac{3}{4} ) _{n-1}}{(n-1)!}r^{2n} \Biggr) \\& \hphantom{f_{1}(r)=}+15 \Biggl( 425\sum_{n=2}^{\infty} \frac{ ( -\frac{1}{2} ) _{n-2}}{(n-2)!}r^{2n}- 624\sum_{n=1}^{\infty} \frac{ ( -\frac{1}{2} ) _{n-1}}{(n-1)!}r^{2n} +192\sum_{n=0}^{\infty} \frac{ ( -\frac{1}{2} ) _{n}}{(n!}r^{2n} \Biggr) \\& \hphantom{f_{1}(r)=}-50 \Biggl( \sum_{n=2}^{\infty} \frac{ ( -\frac{1}{4} ) _{n-2}}{(n-2)!}r^{2n}- 96\sum_{n=1}^{\infty} \frac{ ( -\frac{1}{4} ) _{n-1}}{(n-1)!}r^{2n} +96\sum_{n=0}^{\infty} \frac{ ( -\frac{1}{4} ) _{n}}{n!}r^{2n} \Biggr) \\& \hphantom{f_{1}(r)} = 6\text{,}144-7\text{,}680r^{2}-105r^{4}+2\text{,}592\sum _{n=2}^{\infty}\frac{ ( n-\frac{7}{2} ) ( -\frac{3}{4} ) _{n-1}}{n!}r^{2n} \\& \hphantom{f_{1}(r)=}-15\sum_{n=2}^{\infty}\frac{ ( 7n^{2}-367n-720 ) ( -\frac{1}{2} ) _{n-2}}{n!}r^{2n} \\& \hphantom{f_{1}(r)=}-50\sum_{n=2}^{\infty}\frac{ ( n^{2}-121n+270 ) ( -\frac{1}{4} ) _{n-2}}{n!}r^{2n} \\& \hphantom{f_{1}(r)} = 6\text{,}144-7\text{,}680r^{2}+11\text{,}248r^{4}-1\text{,}944\sum _{n=3}^{\infty}\frac{ ( n-\frac{7}{2} ) ( \frac{1}{4} ) _{n-2}}{n!}r^{2n} \\& \hphantom{f_{1}(r)=}+\frac{15}{2}\sum_{n=3}^{\infty} \frac{ ( 7n^{2}-367n-720 ) ( \frac{1}{2} ) _{n-3}}{n!}r^{2n} \\& \hphantom{f_{1}(r)=}+\frac{25}{2}\sum_{n=3}^{\infty} \frac{ ( n^{2}-121n+270 ) ( \frac{3}{4} ) _{n-3}}{n!}r^{2n}, \\& 16f_{2}(r)-f_{1}(r) \\& \quad =1\text{,}536r^{2}-1\text{,}248r^{4}- \frac{25}{2}\sum_{n=3}^{\infty} \frac{ ( n^{2}-121n+270 ) ( \frac{3}{4} ) _{n-3}}{n!}r^{2n} \\& \quad \quad {}-\frac{15}{2}\sum_{n=3}^{\infty} \frac{ ( 7n^{2}-367n-720 ) ( \frac{1}{2} ) _{n-3}}{n!}r^{2n} +1\text{,}944\sum _{n=3}^{\infty} \frac{ ( n-\frac{7}{2} ) ( \frac{1}{4} ) _{n-2}}{n!}r^{2n} \\& \quad = 1\text{,}536r^{2}-1\text{,}248r^{4}+\sum_{n=3}^{\infty}u_{n}r^{2n}, \end{aligned}$$

where

3.29 $$\begin{aligned}& u_{n}=\frac{972(2n-7)}{n!} \biggl( \frac{1}{4} \biggr) _{n-2} \\& \quad \quad {}-\frac{15(7n^{2}-367n-720)}{2n!} \biggl( \frac{1}{2} \biggr) _{n-3} -\frac{25(n^{2}-121n+270)}{2n!} \biggl( \frac{3}{4} \biggr) _{n-3}, \\& 1-\frac{2}{\pi}\mathcal{E}(r) \\& \quad =-\sum_{n=1}^{\infty} \frac{ ( -\frac{1}{2} ) _{n} ( \frac{1}{2} ) _{n}}{(n!)^{2}}r^{2n} =\sum _{n=1}^{\infty} \frac{ ( \frac{1}{2} ) _{n-1} ( \frac{1}{2} ) _{n}}{2(n!)^{2}}r^{2n}, \\& 16f_{2}(r) \biggl( 1-\frac{2}{\pi}\mathcal{E}(r) \biggr) \\& \quad = 8 \bigl( 625r^{4}-384r^{2}+384 \bigr) \sum _{n=1}^{\infty}\frac{ ( \frac{1}{2} ) _{n-1} ( \frac{1}{2} ) _{n}}{(n!)^{2}}r^{2n} \\& \quad = 1\text{,}536r^{2}-1\text{,}248r^{4}+\sum_{n=3}^{\infty}v_{n}r^{2n}, \end{aligned}$$

where

3.30 $$ v_{n}=\frac{8p_{0}(n)}{(n!)^{2}} \biggl( \frac{1}{2} \biggr) _{n-3} \biggl( \frac{1}{2} \biggr) _{n-2} $$

and $p_{0}(n)$ is defined by (3.9).

3.31 $$\begin{aligned}& J\bigl(r^{\prime}\bigr)={} \frac{(51{r^{\prime}}^{2}+20r^{\prime}\sqrt {r^{\prime}}+50r^{\prime}+20\sqrt{r^{\prime}}+51) (5r^{\prime}-2\sqrt{r^{\prime}}+5)(25{r^{\prime}}^{2}-46r^{\prime }+25)}{16(5r^{\prime}+2\sqrt{r^{\prime}}+5)(5r^{\prime}-2\sqrt {r^{\prime}}+5)(25{r^{\prime}}^{2}-46r^{\prime}+25)} \\& \hphantom{J\bigl(r^{\prime}\bigr) }= \frac{f_{1}(r)}{16f_{2}(r)}, \\& F(r)=\frac{16f_{2}(r)-f_{1}(r)}{16f_{2}(r) ( 1-\frac{2}{\pi}\mathcal {E}(r) ) } =\frac{P(x)+\sum_{n=2}^{\infty}u_{n+1}x^{n}}{P(x)+\sum _{n=2}^{\infty}v_{n+1}x^{n}}. \end{aligned}$$

It follows from (3.9), (3.29), (3.30) and elaborated computations that

3.32 $$\begin{aligned}& \frac{u_{3}}{v_{3}}=1, \end{aligned}$$

3.33 $$\begin{aligned}& u_{n+1}-\frac{v_{n+1}}{v_{n}}u_{n}=-\frac{15(2n-5)}{8(n+1)p_{0}(n)(n+1)!}w_{n}, \end{aligned}$$

where $w_{n}$ is defined by (3.8).

It is not difficult to verify that

3.34 $$ p_{0}(n)=625n^{4}-2\text{,}018n^{3}+3\text{,}985n^{2}-4\text{,}032n+1\text{,}080>0 $$

for all $n\geq3$.

From Lemma 3.3, (3.30), (3.33) and (3.34) we know that

3.35 $$ v_{n}>0 $$

for all $n\geq3$, and the sequence $\{u_{n}/v_{n}\}_{n=3}^{\infty}$ is decreasing.

Equation (3.25) implies that

3.36 $$ P(x)>0 $$

for $x\in(0, 1)$, and $P(x)$ is decreasing on $(0, 1)$.

It follows from Theorem 2.1, (3.31) and (3.32) together with the monotonicity of the sequence $\{u_{n}/v_{n}\} _{n=3}^{\infty}$ and the function $P(x)$ on $(0, 1)$ that the function $F(r)$ is strictly decreasing on $(0, 1)$ and

3.37 $$ \lim_{r\rightarrow1^{-}}F(r)< F(r)< \lim_{r\rightarrow0^{+}}F(r) $$

for all $r\in(0, 1)$.

Note that (3.24), (3.28) and (3.31) lead to the conclusion that

3.38 $$ \lim_{r\rightarrow1^{-}}F(r)=\frac{1-J(0^{+})}{1-2\mathcal {E}(1^{-})/\pi}=\frac{1-51/80}{1-2/\pi}= \frac{29\pi}{80(\pi-2)}, \qquad\lim_{r\rightarrow0^{+}}F(r)=1. $$

Therefore, Theorem 3.4 follows from (3.28), (3.37) and (3.38). □

### Remark 3.5

Let

3.39 $$\begin{aligned}& \lambda_{1}(r)=\frac{\pi}{2}S_{11/4,7/4}\bigl(1, r^{\prime}\bigr), \qquad\mu_{1}(r)=\frac{11}{7}S_{11/4,7/4} \bigl(1, r^{\prime}\bigr), \end{aligned}$$

3.40 $$\begin{aligned}& \lambda_{2}(r)=\frac{25}{16}S_{5/2,2}\bigl(1, r^{\prime}\bigr), \qquad\mu_{2}(r)=\frac{\pi}{2}S_{5/2,2} \bigl(1, r^{\prime}\bigr), \end{aligned}$$

3.41 $$\begin{aligned}& \lambda(r)=\frac{40(\pi-2)}{29}J\bigl(r^{\prime}\bigr)-\frac{51\pi-160}{58}, \qquad\mu(r)=\frac{\pi}{2}J\bigl(r^{\prime}\bigr), \end{aligned}$$

where $J(r^{\prime})$ is defined by (3.24). Then simple computations lead to

3.42 $$\begin{aligned}& \lambda_{1}\bigl(1^{-}\bigr)=\frac{7\pi}{22}=0.999597 \ldots, \qquad\mu_{1}\bigl(0^{+}\bigr)= \frac{11}{7}=1.571428\ldots, \end{aligned}$$

3.43 $$\begin{aligned}& \lambda_{2}\bigl(0^{+}\bigr)=\frac{25}{16}=1.5625, \qquad\mu_{2}\bigl(1^{-}\bigr)=\frac{2\pi}{5}=1.256637 \ldots, \end{aligned}$$

3.44 $$\begin{aligned}& \lambda\bigl(0^{+}\bigr)=\frac{\pi}{2}=\mu\bigl(0^{+} \bigr)=1.5707963\ldots, \end{aligned}$$

3.45 $$\begin{aligned}& \lambda\bigl(1^{-}\bigr)=1, \qquad\mu\bigl(1^{-}\bigr)= \frac{51\pi}{160}=1.00138\ldots. \end{aligned}$$

From (3.3), (3.4), (3.23) and (3.39)-(3.45) we clearly see that there exists small enough $\delta\in(0, 1)$ such that the lower bound given in (3.23) for $\mathcal{E}(r)$ is better than the lower bound given in (3.3) for $r\in(\delta, 1-\delta)$, the lower bound given in (3.23) for $\mathcal{E}(r)$ is better than the lower bound given in (3.4) for $r\in(0, \delta)$, the upper bound given in (3.23) for $\mathcal{E}(r)$ is better than the upper bound given in (3.3) for $r\in(0, \delta)$, and the upper bound given in (3.23) for $\mathcal{E}(r)$ is better than the upper bound given in (3.4) for $r\in(\delta, 1-\delta)$.

### Corollary 3.6

*Let* $J(r^{\prime})$ *be defined by* (3.24). *Then the double inequality*

3.46 $$ \frac{\pi}{2}J\bigl(r^{\prime}\bigr)- \biggl( \frac{51\pi}{160}-1 \biggr) < \mathcal{E}(r)< \frac{\pi}{2}J\bigl(r^{\prime}\bigr) $$

*holds for all* $r\in(0, 1)$.

### Proof

Let $F(r)$ be defined by (3.28) and

3.47 $$ A(r)=J\bigl(r^{\prime}\bigr)-\frac{2}{\pi}\mathcal{E}(r). $$

Then we clearly see that

3.48 $$\begin{aligned}& A\bigl(0^{+}\bigr)=J\bigl(1^{-}\bigr)-\frac{2}{\pi} \mathcal{E}\bigl(0^{+}\bigr)=0, \qquad A\bigl(1^{-}\bigr)=J \bigl(0^{+}\bigr)-\frac{2}{\pi}\mathcal{E}\bigl(1^{-} \bigr)=\frac{51\pi-160}{80\pi}, \end{aligned}$$

3.49 $$\begin{aligned}& A(r)= \biggl[ 1-\frac{2}{\pi}\mathcal{E}(r) \biggr] \biggl[ 1- \frac{1-J(r^{\prime})}{1-\frac{2}{\pi}\mathcal{E}(r)} \biggr] = \biggl[ 1-\frac{2}{\pi}\mathcal{E}(r) \biggr] \bigl[1-F(r)\bigr]. \end{aligned}$$

From (3.49) and the proof of Theorem 3.4 we know that $F(r)$ is strictly decreasing on $(0, 1)$ and $A(r)$ is strictly increasing on $(0, 1)$. Therefore, inequality (3.46) follows from (3.47) and (3.48) together with the monotonicity of $A(r)$ on the interval $(0, 1)$. □

### Corollary 3.7

*Let* $J(r^{\prime})$ *be defined by* (3.24). *Then the double inequality*

3.50 $$ \frac{80}{51}J\bigl(r^{\prime}\bigr)< \mathcal{E}(r)< \frac{\pi}{2}J \bigl(r^{\prime}\bigr) $$

*holds for all* $r\in(0, 1)$.

### Proof

Let $A(r)$ be defined by (3.47) and

3.51 $$ B(r)=\frac{J(r^{\prime})}{\frac{2}{\pi}E(r)}. $$

Then we clearly see that

3.52 $$\begin{aligned}& B\bigl(0^{+}\bigr)=\frac{J(1^{-})}{\frac{2}{\pi}E(0^{+})}=1, \qquad B\bigl(1^{-} \bigr)=\frac{J(0^{+})}{\frac{2}{\pi}E(1^{-})}=\frac{51\pi}{160}, \end{aligned}$$

3.53 $$\begin{aligned}& B(r)=\frac{1}{\frac{2}{\pi}\mathcal{E}(r)} \biggl[ J\bigl(r^{\prime }\bigr)-\frac{2}{\pi} \mathcal{E}(r) \biggr] +1=\frac{A(r)}{\frac{2}{\pi}\mathcal{E}(r)}+1. \end{aligned}$$

From (3.53) and the proof of Corollary 3.6 we know that both $A(r)$ and $B(r)$ are strictly increasing on $(0, 1)$. Therefore, inequality (3.50) follows from (3.51) and (3.52) together with the monotonicity of $B(r)$ on the interval $(0, 1)$. □

### Remark 3.8

From Corollaries 3.6 and 3.7 we have

$$ \biggl\vert \mathcal{E}(r)-\frac{\pi}{2}J\bigl(r^{\prime}\bigr) \biggr\vert < \frac{51\pi}{160}-1=0.001382\ldots,\qquad\biggl\vert \frac{\mathcal{E}(r)-\frac{\pi}{2}J(r^{\prime})}{\mathcal{E}(r)} \biggr\vert < \frac{51\pi}{160}-1=0.001382\ldots\,. $$

for all $r\in(0, 1)$, which implies that both the absolute and relative errors using $\pi J(r^{\prime})/2$ to approximate $\mathcal{E}(r)$ are less than 0.14%.

## Conclusions

In this paper, we find a monotonicity rule for the function $[P(x)+\sum _{n=n_{0}}^{\infty}a_{n}x^{n}]/[P(x)+\sum_{n=n_{0}}^{\infty }b_{n}x^{n}]$. As applications, we present new bounds for the complete elliptic integral $\mathcal{E}(r)=\int _{0}^{\pi/2}\sqrt{1-r^{2}\sin^{2}t}\,dt$ ($0< r<1$) of the second kind, and we show that our bounds are sharper than the previously known bounds for some $r\in(0, 1)$.
